# Identification of a 170-kDa protein over-expressed in lung cancers

**DOI:** 10.1054/bjoc.2001.1828

**Published:** 2001-06

**Authors:** R Pincheira, Q Chen, J-T Zhang

**Affiliations:** Department of Pharmacology and Toxicology, Indiana University Cancer Center, Walther Oncology Center, Indiana University School of Medicine, Indianapolis, IN 46202, USA

**Keywords:** lung cancer, translation initiation, cell growth control, eIF3, p170

## Abstract

Lung cancer is the leading cause for cancer death in both male and female populations. Although many molecular markers for lung cancer have been developed and useful for early detection of lung cancer, their function remains unknown. In this paper, we report our findings that a 170-kDa protein (p170) is over-expressed in all types of human lung cancers compared with normal tissues and it is identified as a subunit of translation initiation factor eIF3 by cDNA cloning. Translation initiation factors are a family of proteins that promote the initiation step of protein synthesis and are regulators of cell growth at the translational level. Further studies showed that p170 mRNA is ubiquitously expressed with higher levels in adult proliferating tissues (e.g. bone marrow) and tissues during development (e.g. fetal tissues). This study suggests that p170 and eIF3 may be important factors for cell growth, development, and tumorigenesis. © 2001 Cancer Research Campaign http://www.bjcancer.com


*British Journal of Cancer* (2001) **84**(11), 1520–1527
© 2001 Cancer Research Campaign
doi: 10.1054/ bjoc.2001.1828, available online at http://www.idealibrary.com on IDEAL®

### Identification of a 170-kDa protein over-expressed in lung cancers

**R Pincheira, Q Chen and J-T Zhang**

Department of Pharmacology and Toxicology, Indiana University Cancer Center, Walther Oncology Center, Indiana University School of Medicine, Indianapolis, IN 46202, USA

**Summary** Lung cancer is the leading cause for cancer death in both male and female populations. Although many molecular markers for lung cancer have been developed and useful for early detection of lung cancer, their function remains unknown. In this paper, we report our findings that a 170-kDa protein (p170) is over-expressed in all types of human lung cancers compared with normal tissues and it is identified as a subunit of translation initiation factor eIF3 by cDNA cloning. Translation initiation factors are a family of proteins that promote the initiation step of protein synthesis and are regulators of cell growth at the translational level. Further studies showed that p170 mRNA is ubiquitously expressed with higher levels in adult proliferating tissues (e.g. bone marrow) and tissues during development (e.g. fetal tissues). This study suggests that p170 and eIF3 may be important factors for cell growth, development, and tumorigenesis. © 2001 Cancer Research Campaign http://www.bjcancer.com

**Keywords**: lung cancer; translation initiation; cell growth control, eIF3; p170

Lung cancer is the leading cause for cancer death in both male and female populations (American Cancer Society, *Cancer Facts and Figures* – 1996). Lung cancer is a disease of heterogeneous histologies and has been divided into 2 major groups: small cell lung cancer (SCLC) and non-small cell lung cancer (NSCLC). NSCLC, accounting for 75% of lung cancers, falls into 3 major types: squamous cell carcinoma (SCC), adenocarcinoma, large cell carcinoma (LCC) and infrequently found carcinoids (Srivastava and Kramer, 1994).

Previous studies indicate that several molecular markers involved in the initiation and progression of lung cancers are useful in predicting prognosis in the early stage of the disease (Kalemkerian, 1994; Stahel, 1995; Zhou et al, 1996; Carbone, 1997). Some marker antigen such as sialoglycoproteins have been found specifically in SCLC while others such as high molecular weight mucins have been found specifically in NSCLC. Although many of these molecular markers have been shown to be useful in early detection of lung cancer, their functions remain unknown.

Regulation of protein synthesis has increasingly been linked to disruption of cell behaviour leading to cancer. It has been shown that over-expression of factors of translation machinery such as elongation factor EF1α (Tatsuka et al, 1992), initiation factors eIF4E (Lazaris-Karatzas et al, 1990), eIF4G (Fukuchi-Shimogori et al, 1997), eIF2α (Donze et al, 1995), and ribosomal protein S3a (Kho et al, 1996), affect cell growth. Expression of eIF4E (De Benedetti and Harris, 1999), the p48 of eIF3 (Asano et al, 1997b), EF1γ (Chi et al, 1992; Lew et al, 1992), and S3a (Pogue-Geile et al, 1991) has also been found deregulated in tumours.

In this paper, we report our finding that a 170-kDa protein was over-expressed in human lung cancers and it was identified as the putative p170 subunit of eIF3 by cDNA cloning. We also show that p170 mRNA is ubiquitously expressed in all tissues tested with high levels in adult proliferating tissues (e.g. bone marrow) and tissues during development (e.g. fetal tissues).

*Received 9 October 2000*
*Revised 22 February 2001*
*Accepted 28 February 2001*

*Correspondence to:* J-T Zhang

### MATERIALS AND METHODS

### Cell lines

HEK 293, HS-294T and Hep G2 were maintained in DMEM containing 10% FBS. HL-60, GC-3 and, MDA-MB-231 cells were maintained in RPMI-1640 medium containing 20% (for HL-60) and 10% (for GC-3 and MDA-MB-231) FBS. IMR-90 was grown in α-MEM containing 10% FBS, 1% L-glutamine, 1% HEPES. SKOV3 cells were maintained in α-MEM supplemented with 15% FBS. Vinblastine was added to the media to a final concentration of 1 μg $ml^{-1}$ for the maintenance of drug-resistant SKOV/VLB cells. The CHO cell lines were maintained in α-MEM medium containing 10% FBS. Colchicine was added to the media to a final concentration of 30 μg $ml^{-1}$ to maintain drug-resistant $CH^rB30$ cells. MCF-7 and its drug-resistant subclone BC-19/3 cells were grown in Eagle's IMEM supplemented with 10% FBS. To maintain the drug resistance of BC-19/3 cells 0.01 μM adriamycin was added to the medium. NIH-3T3 cells was maintained in D-MEM containing 10% Donor Calf Serum and 1% L-glutamine. All cells (except NIH-3T3 that used 10% $CO_2$) were kept at 37˚C in a humidified atmosphere containing 5% $CO_2$.

### Generation of polyclonal antibody AbD

The generation and purification of fusion protein antigen and antibody AbD were performed as previously described (Zhang et al, 1996). Briefly, a cDNA fragment encoding the loop linking putative TM7 and TM8 of hamster Pgp (see Figure 1 for the sequence) was generated by PCR. The PCR product was treated with T4

DNA polymerase and ligated into pGEX-2T vector linearized with Sma I. The positive clone was used to generate fusion protein in YT medium containing 2% glucose, and 100 μg $ml^{-1}$ ampicillin and induced by addition of 0.01 mM IPTG. Fusion protein was purified with glutathione-conjugated Sepharose-4B from the supernatant of the cell lysate and digested with 50 units of thrombin for 5 h at room temperature. The Sepharose beads containing GST and the supernatant containing the Pgp fragment were separated by centrifugation at 500 ***g*** for 30 seconds. The purified Pgp peptide (500 μg) in complete Freund's adjuvant was injected subcutaneously into a female New Zealand white rabbit. 150 μg of purified protein in incomplete Freund's adjuvant was injected as a first boost 30 days after the initial injection, and sera was collected 2–4 times in one month 7 days after each boost. Preimmune serum was collected as a control before the above steps were taken. Affinity purification of the antisera was performed using ImmunoPure immobilization kit from Pierce following the supplier's instructions.

### Isolation of human lymphocytes and macrophages

Human lymphocytes and macrophages were isolated as previously described (Harbeck et al, 1982). Briefly, 10 ml of whole blood were loaded on top of a 45–72% Percoll step gradient and centrifuged at 1500 ***g*** for 25 min at room temperature. After centrifugation, macrophages at the interfaces between plasma and 45% percoll and lymphocytes at the interface between 45% and 54% percoll were collected, centrifuged and washed 2 times with PBS. Final cell pellets were resuspended in hypotonic lysis buffer (10 mM KCl, 1.5 mM $MgCl_2$, 10 mM Tris/HCl, pH 7.4, 2 mM PMSF) and membranes were isolated for Western blot analysis.

### Preparation of membrane fractions from human tissues

Crude membranes were isolated from discarded human tissues using methods as previously described (Riordan and Ling, 1979). Briefly, 0.5 *g* of tissue was homogenized in 10 ml of hypotonic lysis buffer using a tissumizer. The homogenate was then centrifuged at 4000 ***g*** for 15 min to remove cell debris and nuclei. The supernatants were centrifuged again at 100 000 ***g*** for 60 min to enrich the membrane fraction. The final crude membrane pellet was resuspended in 1 ml STBS (0.25 M sucrose, 10 mM Tris/HCl, pH 7.4, 150 mM NaCl, 2 mM PMSF) and stored at –70˚C.

### Library screening

About 300 000 plaques of a λgt11 cDNA library prepared from HL-60 cell line (a generous gift from Dr Javier Navarro, UTMB, Galveston, Texas) were screened using an affinity-purified AbD probe (at 1:500 dilution) and a picoBlue Immunoscreening detection kit (Stratagene). One positive clone was found in the first round of screening and the recombinant λ DNA of the positive clone was isolated. A cDNA insert of 2.4 kb was recovered by PCR using vector primers (F: 5′-GGTGGCGACGACTCCTG-GAGCCCG-3′ and R: 5′-TTGACACCAGACCAACTGGTAAT-G-3′) and the PCR product was cloned into pGEM-T easy plasmid and sequenced using an automated sequencer. To obtain the full-length cDNA, 5′-RACE PCR was performed using 2 antisense p170-specific primers: GSP1: 5′-TTCATCATCCCCCAGACG-3′ and GSP2: 5′-TTCTCTGTTCCTCGTAAGCGCTCT-3′. A product of 2.3 kb was generated, cloned and sequenced.

### Construction of the full-length cDNA of p170

To construct the full-length sequence of p170, we used the cDNA clones isolated by Johnson et al (1997). The full-length cDNA was constructed as described by Valasek et al (1998). The resulting plasmid carrying the full-length p170 was named pUC19-p170. To engineer p170 into expression vectors, a 4.7-kb insert containing full-length human p170 cDNA was excised from pUC19-p170 by digestion with KpnI/BsaAI and cloned into pGEM-4Z plasmid.

### Peptide neutralization

5 μg of crude membrane fractions from SKOV/VLB cells were separated by SDS-PAGE for Western blot analyses. Different dilutions (from 1:1000 to 1:100 000) of the AbD (0.45 μg $ml^{-1}$) were tested to determine the minimum dilution of AbD needed for a consistently positive detection of p170. The minimal amount of AbD needed for detection was neutralized by overnight pre-incubation with excess amount of synthetic peptide. The antibody-peptide complexes were microfuged at top speed for 15 min and the supernatant was used to probe the Western blot.

### In vitro transcription and translation

In vitro transcription and translation were performed as previously described (Zhang and Ling, 1991). Briefly, full-length p170 in pGEM-4Z plasmid was digested with Afl II, and the linearized plasmid was used as template for in vitro transcription using SP6 RNA polymerase (Promega). In vitro transcripts were added to rabbit reticulocyte lysate to program cell-free translation and the translation products were analysed on SDS-PAGE by Western blot or fluorography.

### Master blot analysis

A probe corresponding to the coding region of p170 (bases 2253–3712) was obtained by digesting cDNA with Acl I and Xho I. The probe was labelled using a random-primed labelling kit (Boehinger-Mannheim). A master blot (Clontech) was pre-hybridized for 30 min at 65˚C, in a quick hybridization solution (Stratagene), followed by hybridization for 90 min at 65˚C in the same solution containing 10 μg $ml^{-1}$ salmon sperm DNA, 2 μg $ml^{-1}$ Cot-I DNA, 10 μg $ml^{-1}$ tRNA, and ~$1.25 \times 10^6$ cpm $ml^{-1}$ probe. The blot were then washed twice in 2X SSC/0.1% SDS at room temperature for 15 min each and once in 0.1X SSC/0.1% SDS at 60˚C for 30 min. The blot was finally exposed to an X-ray film at –70˚C for autoradiography. The signal density was determined using a Scion Image program.

### RESULTS

### Detection of p170 by polyclonal antibody AbD

While studying multidrug resistance (MDR) in cancer cells, a polyclonal antibody (AbD) against a peptide (FSKVVGVFTR-NTDDETKRHDSNLFSLLFLILG) of Chinese hamster P-glycoprotein (*pgp1*) was generated. The affinity-purified AbD was tested for its specificity to Pgp in membrane fractions isolated from hamster and human MDR cell lines using Western blot analyses. In hamster, AbD specifically reacted with a 170-kDa protein of drug-resistant $CH^rB30$, but not of sensitive Aux

© 2001 Cancer Research Campaign

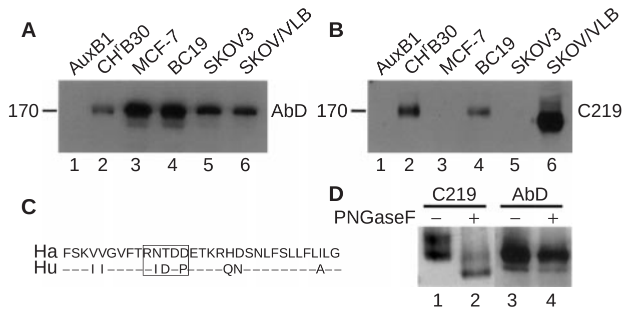

**Figure 1** The difference between p170 and Pgp in human cells. **A** & **B** detection of p170 and Pgp. 5 μg membranes of Aux B1, CH$^{r}$B30, MCF-7, BC19, SKOV3, and SKOV/VLB was probed on a Western blot with affinity-purified polyclonal antibody AbD (panel **A**) or Pgp-specific monoclonal antibody C219 (panel **B**). **C**, comparison between the sequence of Chinese hamster Pgp (top, GenBank accession number M60040) used to generate AbD and that of human Pgp (bottom, GenBank accession number M14758). The boxed sequence indicates the potential epitope for AbD. The dashed line represents the same amino acids between the 2 sequences. **D**, glycosylation status of p170. 5 μg of crude membranes from BC19 cells were digested with (lanes 2 and 4) or without (lanes 1 and 3) endoglycosidase PNGase F followed by Western blot probed with C219 (lanes 1–2) or AbD (lanes 3–4)

B1 cells (Figure 1A, lanes 1 and 2). When the blot was probed with the Pgp-specific monoclonal antibody (mAb) C219 (a gift from Dr Victor Ling) (Georges et al, 1990), the same 170-kDa protein was detected in CH$^{r}$B30 cells (Figure 1B, lanes 1 and 2). Thus, the 170-kDa protein in CH$^{r}$B30 cells detected by AbD is Pgp. In contrast, in human drug-sensitive (MCF-7 and SKOV3) and resistant (BC19 and SKOV/VLB) cancer cell lines AbD reacted with a 170-kDa protein (p170) that was not recognized by mAb C219 (compare lanes 3–6 in Figure 1A and 1B). Note that p170 in drug-resistant SKOV/VLB cells (Figure 1A, lane 6) has a slower mobility than Pgp on SDS-PAGE (Figure 1B, lane 6) (170 kDa versus 160 kDa) and that mAb C219 is known to react with both human and hamster Pgp. The different amino acids between human and hamster Pgp may comprise the AbD epitope which exists in human p170 (Figure 1C). To confirm p170 is not Pgp, we examined the post-translational modification of p170. As shown in Figure 1D, endoglycosidase treatment reduced the size of Pgp detected by C219 (compare lanes 1 and 2). However, the size of p170 detected by AbD was not changed (Figure 1D, lanes 3–4). Thus, p170 is not a glycoprotein and it is different from Pgp.

### Expression of p170 in human cancer cell lines

Because p170 is expressed similarly in both drug-resistant and -sensitive human cancer cells, it is not likely to be related to drug resistance. Thus, we questioned whether p170 expression is related to cell proliferation and/or malignant transformation. To answer this question, we tested 6 more human cancer cell lines (A2780, MDA-MB-231, HS-294T, HepG2, HL-60, GC-3), one normal human lung fibroblast cell line (IMR-90), and normal lymphocytes and macrophages. As shown in Figure 2, p170 expression is elevated in all cancer cell lines as compared to the normal cells using a housekeeping protein calnexin as an internal control. Thus, it is possible that increased p170 expression is related to cell proliferation and/or malignant transformation.

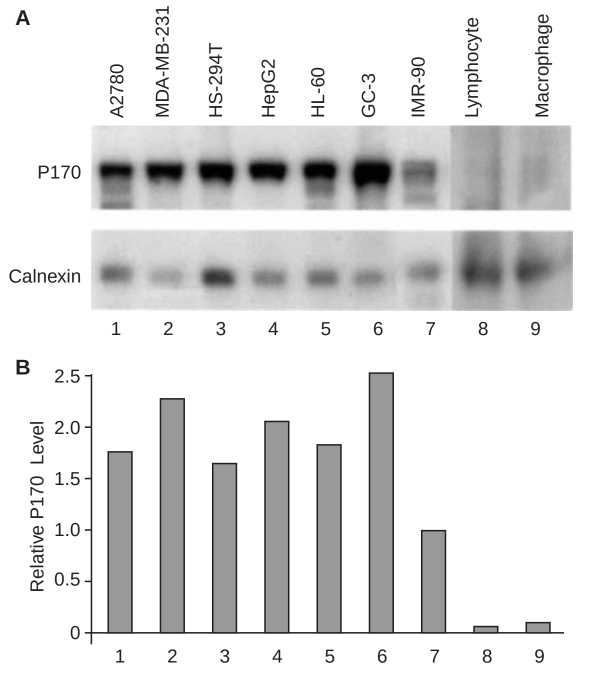

**Figure 2** Expression of p170 in cancer and normal cell lines. Crude membranes were prepared from cell lines of human ovarian cancer A2780 (lane 1), breast cancer MDA-MB-231 (lane 2), melanoma HS-294T (lane 3), hepatoma HepG2 (lane 4), leukaemia HL-60 (lane 5), colon cancer GC-3 (lane 6), normal lung fibroblast IMR-90 (lane 7), normal lymphocytes (lane 8), and normal macrophages (lane 9). 5 μg crude membranes were used for Western blot analysis probed with AbD. Calnexin was used as a control for protein loading. Panel **B** shows p170 expression level normalized to calnexin of panel A as determined using Scion image software

### Expression of p170 in human cancer tissues

To determine whether p170 over-expression is relevant to tumorigenesis, we tested p170 expression in cancer and normal human tissues (obtained from Cooperative Human Tissue Network). Crude membrane fractions from human ovary, kidney, lung, breast, and colon were isolated and analysed by Western blot (Figure 3). We found a significant difference of p170 expression only between lung cancer and its corresponding normal tissue. Lung cancer tissues express a high p170 level whereas normal lung tissues express low and undetectable levels of p170 (Figure 3A,

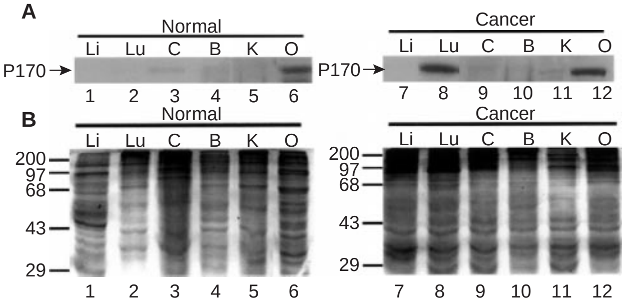

**Figure 3** Expression of p170 in human tissues. **A**, detection of p170 in human tissues. 5 μg crude membranes from normal or cancer human liver (Li), lung (Lu), colon (C), breast (B), kidney (K), and ovary (O) were subjected to Western blot analysis probed with AbD. **B**, Coomassie blue staining of protein profile of membranes of human tissues as shown in (**A**). About 30 μg of proteins each was resolved on a SDS-PAGE and stained with coomassie blue

 *© 2001 Cancer Research Campaign*

**Table 1** Detection of P170 protein in normal and cancer human tissues

| Tissues | Cancer<br>No. of P170(+)/Total[a] | Normal<br>No. of P170(+)/Total[a] |
|---|---|---|
| Kidney | 0/4 | 0/4 |
| Ovary | 4/4 | 4/4 |
| Breast | 1/5 | 0/4 |
| Colon | 1/5 | 0/4 |
| Liver | 1/5 | 0/2 |
| Lung | 15/18 | 1/10 |

[a]Number of cases that show a positive signal of p170 detected by AbD on Western blot over total number of tissues tested.

**Table 2** Correlation between P170 expression and lung cancer histology

| Histology | No. of P170(+)/Total |
|---|---|
| SCLC | 2/2 |
| LCC | 2/3 |
| SCC | 7/7 |
| AC | 4/6 |

**Table 3** Nature of lung cancer tissues tested

| Sample | Age/Sex | Treatment | Differentiation stage[a] | P170[b] |
|---|---|---|---|---|
| SCLC | 60/M | Yes | P | + |
| SCLC | N/A | N/A | P | + |
| LCC | 60/F | No | U | – |
| LCC | 70/M | Yes | P | + |
| LCC | 66/F | No | P | + |
| SCC | 70/M | No | M/P | + |
| SCC | 65/F | Yes | M/P | + |
| SCC | 73/F | N/A | M | + |
| SCC | 75/M | No | P | + |
| SCC | 67/F | No | M | + |
| SCC | 60/M | No | P | + |
| SCC | 47/M | N/A | P | + |
| AC | 73/M | N/A | P | + |
| AC | 68/M | No | P | + |
| AC | 81/F | N/A | M | – |
| AC | 68/M | N/A | D | + |
| AC | 43/M | No | M | – |
| AC | 62/F | Yes | M | + |

[a]The differentiation stage of the cancer tissues are categorized as U (undifferentiated), P (poorly differentiated), M (moderately differentiated), and D (differentiated). [b]P170 expression was detected (+) or not detected (–) using AbD on Western blot.

lanes 2 and 8). P170 expression level was undetectable in other cancer tissues compared to their corresponding normal tissues (Figure 3A, compare lanes 1, 3–6 with lanes 7, 9–12, respectively). It is, however, interesting to note that high expression level of p170 was found in both normal and cancer ovarian tissues.

Table 1 summarizes the results of all human tissues analysed. Of the 18 lung-cancer samples examined, 15 had elevated expression of p170 and only one of the 10 normal corresponding tissues had a detectable level of p170. Only one each of 5 cases of breast, colon and liver tumours has elevated expression of p170. Table 2 shows the correlation between the elevated expression of p170 and the types of lung cancers. Apparently, all types of lung cancers express elevated level of p170. We also analysed clinical parameters such as age, histology and treatment history of the lung cancer patients tested. Table 3 summarizes the nature of human lung tissues used and no apparent correlation was found between p170 expression and the clinical parameters considered.

### Cloning of p170

To identify p170, we cloned p170 cDNA from a λgt-11 cDNA expression library. The full-length cDNA of 4.5 kb encodes a protein of 1382 amino acids (Figure 4A). By searching the GeneBank, we found that the p170 cDNA encodes the 170-kDa subunit of eIF3 (Johnson et al, 1997). Analysis of the deduced amino acid sequence showed that there was a stretch of 5 amino acids RNTDD (underlined in Figure 4) which is the same as in hamster but not in human Pgp (see the boxed sequence in Figure 1C). Thus, it is possible that RNTDD represents the AbD epitope responsible for AbD detection.

The eIF3 p170 cDNA was confirmed by 4 experiments. Firsly, a synthetic peptide (TRNTDDE) was able to inhibit the reactivity of AbD to p170 but not the irrelevant antibody C219 (compare Figure 5A with Figure 5B). Thus, RNTDD may represent AbD epitope and likely AbD reacts with eIF3 p170 (Johnson et al, 1997). Secondly, antibody AbF against the fusion protein generated from eIF3 p170 cDNA (Johnson et al, 1997) detected the same p170 on Western blot detected by AbD (Figure 5C, lanes 1 and 3) as well as with the p170 immunoprecipitated by AbD (Figure 5C, lanes 2 and 4). Thirdly, eIF3 p170 cDNA (Johnson et al, 1997) was translated in rabbit reticulocyte lysate (RRL) and the translation product of 170-kDa reacted strongly with AbD on Western blot (Figure 5D, lanes 1 with 2–5) and by immunoprecipitation (data not shown). Lastly, the higher level of p170 was detected in human lung cancer cell line H1299 than in normal lung fibroblast IMR90 by eIF3 authentic antibodies AbF (Johnson et al, 1997), M116 (Johnson et al, 1997), and eIF3 antiserum (Meyer et al, 1982).

### Amino acid sequence analysis of p170

To examine what important functional domain p170 may have, we searched p170 amino acid sequence against several protein motif databases. Two main interesting motifs were identified with p170 sequence, which have not been reported previously. One is a PCI domain from amino acid 405 to 495 (double-boxed amino acid sequences, Figure 4A and 4B) and the other is spectrin repeats (single-boxed sequences, Figure 4A and 4B). The PCI domain (Hofmann and Bucher, 1998) consists of pure α-helical structures of ~200 amino acid residues generally located at the C-terminus of the protein. It has been found in yeast and mammalian *P*roteasome regulatory components, in plant COP9-complex proteins involved in light signalling and in translation Initiation factor 3 complex subunit p110 and p48 (Hofmann and Bucher, 1998). Because proteins containing this domain are all part of a large multi-protein complex, it is possible that the PCI domain plays a role in protein–protein interaction. The PCI domain in p170 may play an important role in its interaction with eIF4B, p44 and p116 of eIF3. The spectrin repeat is a major constituent of several proteins belonging to the spectrin family of actin-binding proteins. Spectrin is a major component of the membrane cytoskeleton and the spectrin repeat is made up of 3 α-helices separated by 2 loop regions (Pascual et al, 1997). Another interesting domain in the p170 amino acid sequence is a 10 amino acid tandem repeat (in grey, Figure 4). This repeat has been reported previously (Nagase et al,

© 2001 Cancer Research Campaign 

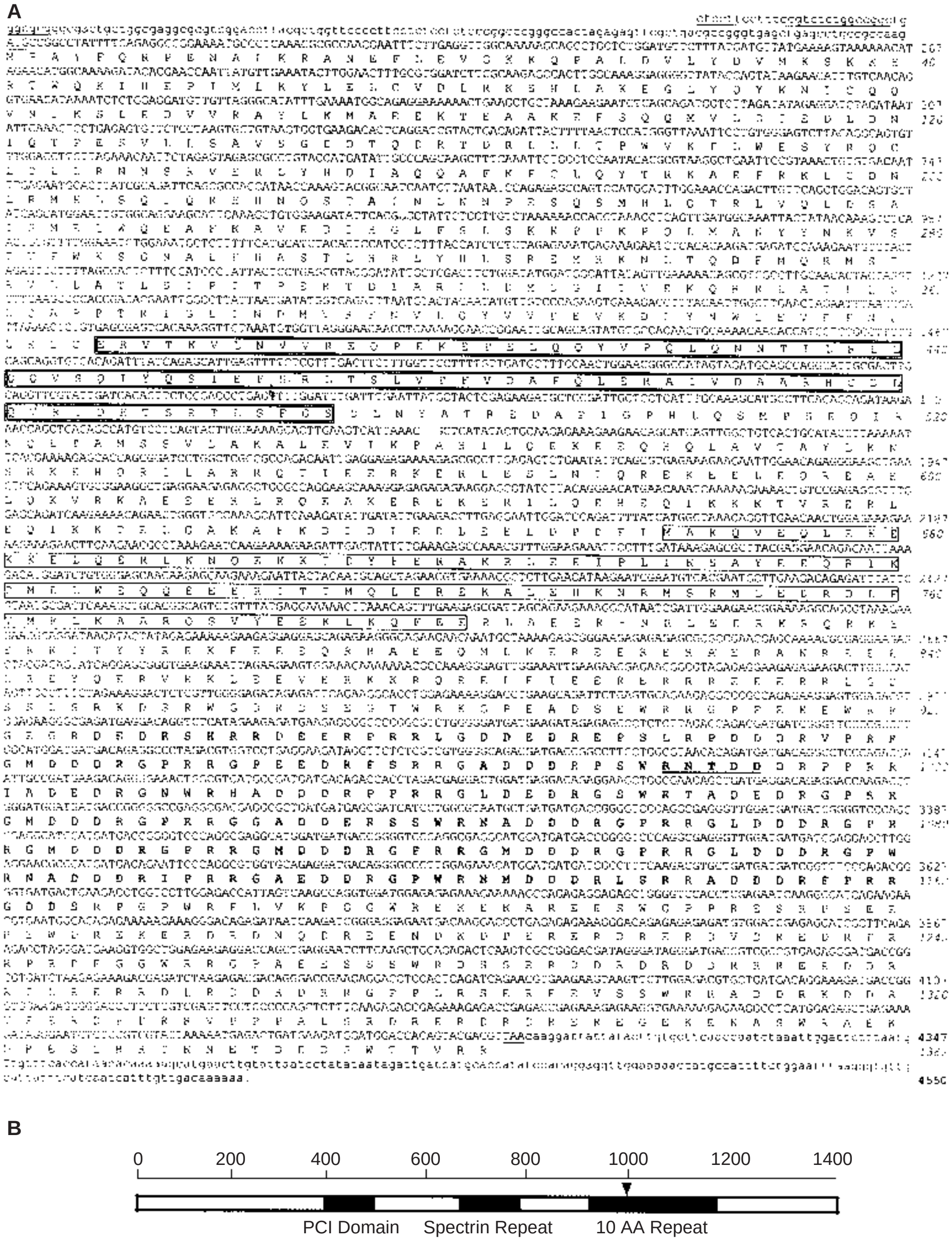

**Figure 4** Sequence of p170. Amino acid sequence is shown in single letter codes and the untranslated regions are shown in small letters. The double- and single-boxed amino acid sequences are the putative PCI domain and the putative spectrin repeat, respectively. The shaded region is the domain of 10 amino acid repeat. AbD epitope RNTDD is shown underlined. The arrowhead in **B** indicates the relative position of AbD epitope

1995; Bachmann et al, 1997; Johnson et al, 1997; Scholler and Kanner, 1997) and was suggested to have a role in the interaction between p170 and eIF4B (Methot et al, 1996).

### Expression of p170 mRNA in human tissues

In the above studies, we showed that the expression of p170 protein was elevated in cancer cell lines and in lung cancer tissues on Western blot. To further understand the expression of p170, we probed an array of RNAs from various normal adult and fetal human tissues with p170 cDNA. As shown in Figure 6B, all tissues expressed p170 mRNA but at different levels. It is interesting to note that the proliferating tissues such as bone marrow (E8), thymus (E5), and digestive tissues (C8, D3) express highest mRNA levels. Also, most fetal tissues express higher level of p170 mRNA than the corresponding adult tissues. For example, the expression level in fetal lung (G7) is ~3 times more than that in adult lung (F2). This observation suggests that higher expression level of p170 is required for cell proliferation and tissue development.

 *© 2001 Cancer Research Campaign*

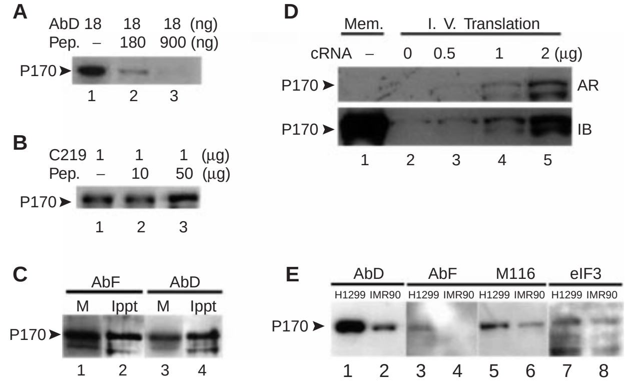

**Figure 5** Confirmation of eIF3 p170 cDNA clone. **A** and **B**, peptide inhibition of AbD and C219 reaction. AbD (**A**) and C219 (**B**) were preincubated without (lane 1) or with epitope peptide (lanes 2 and 3) prior probing a Western blot of SKOV/VLB membranes. **C**, immunoprecipitation of p170. Cell lysates of SKOV/VLB were precipitated by AbD and analysed by Western blot using AbF (lanes 1–2) or AbD (lanes 3–4). Lanes 1 and 3 are control SKOV/VLB membranes. **D**, in vitro translation of p170 cRNA. P170 cRNA was transcribed from p170 cDNA and used to programme cell-free translation in rabbit reticulocyte lysate in the presence of [$^{35}$S]methionine (lanes 2–5). Translation products were then separated by SDS-PAGE for autoradiography (AR) and Western blot (IB) analysis. Lane 1 is the control membrane from SKOV/VLB cells. Note that endogenous p170 in rabbit reticulocyte lysate was not detected by AbD possibly due to lack of AbD epitope in rabbit p170. **E**, Western blot analysis of H1299 and IMR90 cells with eIF3 antibodies. 10-µg proteins from lung cancer cell line H1299 and normal lung fibroblast IMR90 were subjected to Western blot analysis. Antibodies used are AbD, AbF (Johnson et al, 1997), M116 (Johnson et al, 1997), and eIF3 antiserum (Meyer et al, 1982)

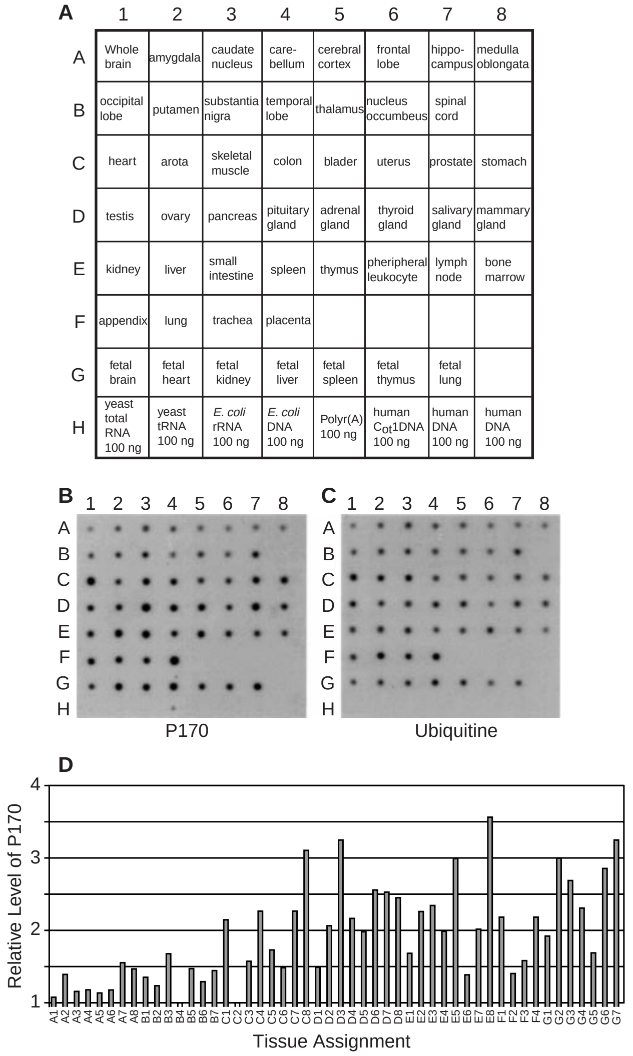

**Figure 6** Master blot of human tissues. A master blot containing total RNAs isolated from various adult and fetal human tissues was probed with p170 (panel **B**) and ubiquitine (panel **C**) probes. Panel **A** indexes the tissues used. Panel **D** represents the relative intensity of p170 in panel B determined using Scion Image and normalized against the corresponding intensity of ubiquitine in panel C, and then normalized against the value of aorta (C2)

### DISCUSSION

In this paper, we report our findings that p170 is over-expressed in human lung cancers and in cultured cancer cell lines compared with normal lung tissues and normal cells, respectively. The p170 protein was identified to be eIF3 p170 by cDNA cloning, characterization, and detection of p170 in human lung cancer cell line using antibodies directed against eIF3 p170 (AbF, M116, and eIF3). The cDNA was confirmed to encode the 170-kDa protein detected in cancer cells by (a) in vitro translation, (b) immunoprecipitation and Western blot with antibody AbF, (c) AbD epitope identification, and (d) detection of p170 over-expressed in human lung cancer cells with authentic eIF3 p170 antibodies. Thus, we conclude that eIF3 p170 is the p170 over-expressed in human lung cancers.

eIF3, a translation initiation factor, consists of 8 or more protein subunits (Asano et al, 1997a). It plays a key role in protein synthesis by interacting and stabilizing the ternary complex of eIF2.GTP.Met-tRNA$_i$ and by helping the binding and stabilization of the Met-tRNA$_i$ on 40S ribosomal subunits (Trachsel et al, 1977; Benne et al; 1979; Asano et al, 1997a). One of the eIF3 subunits, p170 (Johnson et al, 1997), has been demonstrated to interact with other components of the eIF3 such as p44 (Block et al, 1998) and p116 (Methot et al, 1997), and also with eIF4B (Methot et al, 1996) as well as RNA (Block et al, 1998; Buratti et al, 1998). Because of its interaction with RNA and multiple proteins, p170 has been postulated to play an important role in all eIF3 functions (Johnson et al, 1997; Block et al, 1998; Valasek et al, 1998). P170 has also been suggested to play a role in the cap-independent translation (Buratti et al, 1998). However, it has been observed that eIF3 preparations, which essentially lacked p170, did not differ substantially in specific activity from preparations relatively rich in p170 (Meyer et al, 1981). This is supported by a recent study (Chaudhuri et al, 1997) which showed that purified eIF3 complex lacking p170 was able to stimulate the binding of the ternary complex to 40S ribosome. This observation indicates that p170 may not be a true subunit of eIF3, but only interact with the eIF3 complex in a discrete step in the translation initiation process. P170 could also, under specific conditions, dissociate from eIF3 complex and exert other specific function(s) (Chaudhuri et al, 1997; Valasek et al, 1998).

Previously, a p170 homologous protein (p150) cloned from mouse has been found over-expressed in human breast, cervical and esophageal cancers (Bachmann et al, 1997; Dellas et al, 1998; Chen and Burger, 1999). We also analysed p170 expression in human breast tissues. However, p170 over-expression was found in only one of the 5 samples examined (Table 1). The cause for the difference between this study and that of Bachmann et al (1997) is not known. However, it is possible that different isoforms of p170 is over-expressed in different cancer tissues.

© 2001 Cancer Research Campaign

Nevertheless, both Bachmann's and our studies suggest that p150/p170 over-expression correlates with cancers in human breast, cervix, esophagus and lung.

In this study, we found no correlation between p170 over-expression and clinical parameters such as age, histology, and treatment history of the lung cancer patients tested. However, a correlation has been found between cancer progression and p150 (mouse homologue of human p170) expression in cervical cancers. The highest p150 level was found at the begining of the malignancy and the level decreased with cancer progression (Dellas et al, 1998). P150 expression has also been found to correlate with the cell differentiation (Bachmann et al, 1997). The more differentiated cells express less p150 or vice versa.

It is unknown why p170 is not detected in the membrane fraction of normal human lung tissues considering that it may be a subunit of eIF3 and its mRNA is expressed in normal tissues. However, it has been shown that p170 could not be detected in many normal human breast tissues (Bachmann et al, 1997). Furthermore, although eIF3 is a ubiquitous and necessary factor for protein synthesis, it has also been shown that p170 may not be necessary for the function of eIF3 (Chaudhuri et al, 1997). P170 may be a protein co-isolated with eIF3 and it may not be constitutively expressed at the protein level. Considering the high GC content (70%) and a potential secondary structure in the 5′-UTR of p170, its expression may be under translational control.

Although it is currently unknown whether over-expression of p170 in human lung cancer is a result or potentially a cause of lung tumorigenesis, it is interesting to note that the p170 mRNA is highly expressed in the rapid proliferating tissues such as bone marrow and thymus and in the developing fetal tissues. Together with the study of mouse homologue p150 (Bachmann et al, 1997), this observation suggests that p170 over-expression may be important for cell growth, proliferation and tumorigenesis. The possibility that a subunit of eIF3 may be involved in lung tumorigenesis is also in agreement with the important role of protein synthesis in cell-growth, differentiation and transformation. eIF3 is a key element in the protein translation initiation step. Through its binding to the 40S ribosomal subunits, eIF3 plays important roles in several different steps of the translation initiation process, including dissociation of ribosome (Trachsel et al, 1977), binding and stabilization of the ternary complex (Trachsel et al, 1977; Benne et al, 1979), and binding of mRNA (Goumans et al, 1980; Naranda et al, 1994; Lamphear et al, 1995; Buratti et al, 1998). It is possible that disruption of the normal function of eIF3 complex by over-expressing one of its subunits such as p170 can lead to abnormal cell growth. Recently, the p40 subunit of eIF3 was also found over-expressed in human breast and prostate cancers (Nupponen et al, 1999).

### ACKNOWLEDGEMENTS

We would like to thank Ms Guichun Wang, Chunping Gu, Alma Altamirano, and Mei Zhang for their technical support. We would also like to thank Drs Guillermo Altenberg, Len Erickson, Julie Horton, Victor Ling, and Javier Navarro for providing cell lines and Drs John Hershey and Keith Johnson for providing eIF3 antibodies. Critical comments on an earlier version of this manuscript from Mr Daniel Rojo, and Drs Ariel Castro, David Ohannesian, Ernest S Han, and Emi Kreklau are greatly appreciated. This work was supported in part by National Institutes of Health Grants CA64539 and GM59475. J-TZ is a recipient of a Career Investigator Award from the American Lung Association.

### REFERENCES

Asano K, Kinzy TG, Merrick WC and Hershey JW (1997a) Conservation and diversity of eukaryotic translation initiation factor eIF3. *J Biol Chem* **272**: 1101–1109

Asano K, Merrick WC and Hershey JW (1997b) The translation initiation factor eIF3-p48 subunit is encoded by int-6, a site of frequent integration by the mouse mammary tumor virus genome. *J Biol Chem* **272**: 23477–23480

Bachmann F, Banziger R and Burger MM (1997) Cloning of a novel protein overexpressed in human mammary carcinoma. *Cancer Res* **57**: 988–994

Benne R, Brown-Luedi ML and Hershey JW (1979) Protein synthesis initiation factors from rabbit reticulocytes: purification, characterization, and radiochemical labeling. *Methods Enzymol* **60**: 15–35

Block KL, Vornlocher HP and Hershey JW (1998) Characterization of cDNAs encoding the p44 and p35 subunits of human translation initiation factor eIF3. *J Biol Chem* **273**: 31901–31908

Buratti E, Tisminetzky S, Zotti M and Baralle FE (1998) Functional analysis of the interaction between HCV 5′UTR and putative subunits of eukaryotic translation initiation factor eIF3. *Nucleic Acids Res* **26**: 3179–3187

Carbone DP (1997) The biology of lung cancer. *Seminars Oncol* **24**: 388–401

Chaudhuri J, Chakrabarti A and Maitra U (1997) Biochemical characterization of mammalian translation initiation factor 3 (eIF3). Molecular cloning reveals that p110 subunit is the mammalian homologue of Saccharomyces cerevisiae protein Prt1. *J Biol Chem* **272**: 30975–30983

Chen G and Burger MM (1999) p150 expression and its prognostic value in squamous-cell carcinoma of the esophagus. *Int J Cancer* **84**: 95–100

Chi K, Jones DV and Frazier ML (1992) Expression of an elongation factor I gamma-related sequence in adenocarcinomas of the colon. *Gastroenterology* **103**: 98–102

De Benedetti A and Harris AL (1999) eIF4E expression in tumours: its possible role in progression of malignancies. *Int J Biochem Cell Biol* **31**: 59–72

Dellas A, Torhorst J, Bachmann F, Banziger R, Schultheiss E and Burger MM (1998) Expression of p150 in cervical neoplasia and its potential value in predicting survival. *Cancer* **83**: 1376–1383

Donze O, Jagus R, Koromilas AE, Hershey JW and Sonenberg N (1995) Abrogation of translation initiation factor eIF-2 phosphorylation causes malignant transformation of NIH 3T3 cells. *EMBO J* **14**: 3828–3834

Fukuchi-Shimogori T, Ishii I, Kashiwagi K, Mashiba H, Ekimoto H and Igarashi K (1997) Malignant transformation by overproduction of translation initiation factor eIF4G. *Cancer Res* **57**: 5041–5044

Georges E, Bradley G, Gariepy J and Ling V (1990) Detection of P-glycoprotein isoforms by gene-specific monoclonal antibodies. *Proc Natl Acad Sci* **87**: 152–156

Goumans H, Thomas A, Verhoeven A, Voorma HO and Benne R (1980) The role of eIF-4C in protein synthesis initiation complex formation. *Biochim Biophys Acta* **608**: 39–46

Hann SR (1994) Regulation and function of non-AUG-initiated proto-oncogenes. *Biochim* **76**: 880–886

Harbeck RJ, Hoffman AA, Redecker S, Biundo T and Kurnick J (1982) The isolation and functional activity of polymorphonuclear leukocytes and lymphocytes separated from whole blood on a single percoll density gradient. *Clin Immunol Immunopathol* **23**: 682–690

Hofmann K and Bucher P (1998) The PCI domain: a common theme in three multiprotein complexes. *Trends Biochem Sci* **23**: 204–205

Johnson KR, Merrick WC, Zoll WL and Zhu Y (1997) Identification of cDNA clones for the large subunit of eukaryotic translation initiation factor 3. Comparison of homologues from human, Nicotiana tabacum, Caenorhabditis elegans, and Saccharomyces cerevisiae. *J Biol Chem* **272**: 7106–7113

Kalemkerian GP (1994) Biology of lung cancer. *Curr Opinion Oncol* **6**: 147–155

Kho CJ, Wang Y and Zarbl H (1996) Effect of decreased fte-1 gene expression on protein synthesis, cell growth, and transformation. *Cell Growth Differentiation* **7**: 1157–1166

Lamphear BJ, Kirchweger R, Skern T and Rhoads RE (1995) Mapping of functional domains in eukaryotic protein synthesis initiation factor 4G (eIF4G) with picornaviral proteases. Implications for cap-dependent and cap-independent translational initiation. *J Biol Chem* **270**: 21975–22183

Lazaris-Karatzas A, Montine KS and Sonenberg N (1990) Malignant transformation by a eukaryotic initiation factor subunit that binds to mRNA 5′ cap. *Nature* **345**: 544–547

Lew Y, Jones DV, Mars WM, Evans D, Byrd D and Frazier ML (1992) Expression of elongation factor-1 gamma-related sequence in human pancreatic cancer. *Pancreas* **7**: 144–152

Methot N, Song MS and Sonenberg N (1996) A region rich in aspartic acid, arginine, tyrosine, and glycine (DRYG) mediates eukaryotic initiation factor

 *© 2001 Cancer Research Campaign*

4B (eIF4B) self-association and interaction with eIF3. *Mol Cell Biol* **16**: 5328–5334

Methot N, Rom E, Olsen H and Sonenberg N (1997) The human homologue of the yeast Prt1 protein is an integral part of the eukaryotic initiation factor 3 complex and interacts with p170. *J Biol Chem* **272**: 1110–1116

Meyer LJ, Brown-Luedi ML, Corbett S, Tolan DR and Hershey JW (1981) The purification and characterization of multiple forms of protein synthesis eukaryotic initiation factors 2, 3, and 5 from rabbit reticulocytes. *J Biol Chem* **256**: 351–356

Meyer LJ, Milburn SC and Hershey JW (1982) Immunochemical characterization of mammalian protein synthesis initiation factors. *Biochemistry* **21**: 4206–4212

Nagase T, Seki N, Tanaka A, Ishikawa K and Nomura N (1995) Prediction of the coding sequences of unidentified human genes. IV. The coding sequences of 40 new genes (KIAA0121-KIAA0160) deduced by analysis of cDNA clones from human cell line KG-1 (supplement). *DNA Res* **2**: 199–210

Naranda T, MacMillan SE and Hershey JW (1994) Purified yeast translational initiation factor eIF-3 is an RNA-binding protein complex that contains the PRT1 protein. *J Biol Chem* **269**: 32286–32292

Nupponen NN, Porkka K, Kakkola L, Tanner M, Persson K, Borg A, Isola J and Visakorpi T (1999) Amplification and overexpression of p40 subunit of eukaryotic translation initiation factor 3 in breast and prostate cancer. *Am J Pathol* **154**: 1777–1783

Pascual J, Castresana J and Saraste M (1997) Evolution of the spectrin repeat. *Bioessays* **19**: 811–817

Pogue-Geile K, Geiser JR, Shu M, Miller C, Wool IG, Meisler AI and Pipas JM (1991) Ribosomal protein genes are overexpressed in colorectal cancer: isolation of a cDNA clone encoding the human S3 ribosomal protein. *Mol Cell Biol* **11**: 3842–3849

Riordan JR and Ling V (1979) Purification of P-glycoprotein from plasma membrane vesicles of Chinese hamster ovary cell mutants with reduced colchicine permeability. *J Biol Chem* **254**: 12701–12705

Scholler JK and Kanner SB (1997) The human p167 gene encodes a unique structural protein that contains centrosomin A homology and associates with a multicomponent complex. *DNA Cell Biol* **16**: 515–531

Srivastava S and Kramer B (1994) Genetics of lung cancer: Implications for early detection and prevention. In *Lung cancer* Hansen HH (ed) pp. 91–110. Kluwer Academic Publishers: Norwell

Stahel RA (1995) Monoclonal antibodies to membrane antigens of lung cancer. In *Lung Cancer* D.D, C. (ed) pp. 191–212. Arnold: Lung Cancer

Tatsuka M, Mitsui H, Wada M, Nagata A, Nojima H and Okayama H (1992) Elongation factor-1 alpha gene determines susceptibility to transformation. *Nature* **359**: 333–336

Trachsel H, Erni B, Schreier MH and Staehelin T (1977) Initiation of mammalian protein synthesis. II. The assembly of the initiation complex with purified initiation factors. *J Mol Biol* **116**: 755–767

Valasek L, Trachsel H, Hasek J and Ruis H (1998) Rpg1, the Saccharomyces cerevisiae homologue of the largest subunit of mammalian translation initiation factor 3, is required for translational activity. *J Biol Chem* **273**: 21253–21260

Willis AE (1999) Translational control of growth factor and proto-oncogene expression. *Int J Biochem Cell Biol* **31**: 73–86

Zhang JT and Ling V (1991) Study of membrane orientation and glycosylated extracellular loops of mouse P-glycoprotein by in vitro translation. *J Biol Chem* **266**: 18224–18232

Zhang M, Wang G, Shapiro A and Zhang JT (1996) Topological folding and proteolysis profile of P-glycoprotein in membranes of multidrug-resistant cells: implications for the drug-transport mechanism. *Biochemistry* **35**: 9728–9736

Zhou J, Mulshine JL, Unsworth EJ, Scott FM, Avis IM, Vos MD and Treston AM (1996) Purification and characterization of a protein that permits early detection of lung cancer. Identification of heterogeneous nuclear ribonucleoprotein-A2/B1 as the antigen for monoclonal antibody 703D4. *J Biol Chem* **271**: 10760–10766

*© 2001 Cancer Research Campaign*
